# Stuck fragment of totally implantable central venous access ports during removal: risk factor analysis in children

**DOI:** 10.1186/s12893-021-01271-7

**Published:** 2021-06-02

**Authors:** Hanna Jung, Joon Yong Cho, Yangki Seok, Youngok Lee

**Affiliations:** 1 Department of Thoracic and Cardiovascular Surgery, Kyungpook National University Hospital, Kyungpook National University School of Medicine, Daegu, 41944 Republic of Korea; 2 Department of Thoracic and Cardiovascular Surgery, Soonchunhyang University Gumi Hospital, Gumi, 39371 Republic of Korea

**Keywords:** Child, Risk factor, Vascular access ports

## Abstract

**Background:**

Totally implantable central venous access ports (TICVAPs) have increasingly been used in pediatric patients because they provide reliable venous access. However, many complications associated with TICVAPs have been reported. Here, we aimed to analyze the risk factors of stuck fragment of TICVAPs during removal in children and recommend the appropriate periods of use or exchange.

**Methods:**

We retrospectively reviewed the medical records of 121 patients, including 147 cases of TICVAP insertion, between January 2010 and July 2020.

**Results:**

Among these, 98 cases in 72 patients involved of TICVAP removal, with 8 patients having had incomplete TICVAP removal resulting in a stuck fragment of the catheter in the central venous system (Group S). All Group S patients were male and had acute leukemia, and their TICVAPs were used for chemotherapy. Compared with the complete removal group (Group N), stuck fragment in Group S were significantly found in patients diagnosed with acute leukemia than those with other diagnoses (p < 0.001). Indwelling duration and body weight change during TICVAP indwelling were significantly longer and larger in Group S, respectively (p < 0.001). In multivariate logistic regression analysis, indwell duration (odds ratio [OR], 1.13; 95% confidence interval [Cl] 1.02–1.37, p = 0.10), body weight change during indwell (OR, 1.00; 95% Cl 0.83–1.18, p = 0.97), and platelet count at TICVAP insertion (OR, 0.98; 95% Cl 0.95–0.99; p = 0.48) showed an increased trend of risk for a stuck catheter.

**Conclusions:**

We suggest prophylactic catheter exchange before indwell duration of 46 months (area under the curve [AUC], 0.949; 95% Cl 0.905–0.993) and body weight change up to 9.9 kg (AUC, 0.903; 95% Cl 0.840–0.966) to prevent a catheter from becoming stuck, especially in children with rapidly growing acute leukemia. Management of a stuck fragment remains controversial in asymptomatic patients, and we suggest careful, close observation rather than aggressive and invasive treatment.

## Background

A totally implantable central venous access port (TICVAP), also known as a chemoport or cancer port, is a small reservoir connected to a venous catheter positioned in the subcutaneous or muscle layer. Long venous access devices have been used for various indications such as fluid or intravenous drug administration, blood transfusions, or instillation of toxic chemotherapeutic drugs in oncology. In particular, TICVAPs are necessary in many chronically ill pediatric patients because of infusion of long-term intravenous therapy. With a TICVAP, there is no need for children to undergo multiple peripheral venipunctures, which may injure the peripheral veins and cause psychological and emotional trauma in children. Moreover, the peripheral vein has a risk of extravasation of toxic chemotherapeutic drugs.

In 1982, Niederhubur and his colleague first performed the implantation of subcutaneous tunneled central venous ports. Since then, TICVAPs have increasingly been used in the field of pediatric oncology to provide reliable vascular access [1]. Currently, TICVAPs promote repeat and/or prolonged intravenous therapy for children [2]. After the completion of treatment, the ports can be easily removed. Despite having many advantages, TICVAPs also have several complications, such as infection, thrombosis, catheter disconnection, or fracture with migration and stuck catheters [2–5]. The stuck catheter complication is rare, and research on this issue has been scarce. The catheter portion of the TICVAP can be firmly adhered and stuck to the vessel wall, making the removal of the entire catheter difficult. In such circumstances, excessive pulling force can fracture the catheter, leaving a portion retained in the vessel. The incidence of retained fragment of central venous catheters due to unsuccessful removal is 0.4–2% of all line removals [3]. However, the incidence of stuck fragment of TICVAPs remains unknown.

Many studies have analyzed complications associated with TICVAPs. However, few reports have been published on adherent tunneled, retained fragments, or stuck catheters of TICVAPs. The purpose of this retrospective study was to analyze the risk factors of stuck fragment of TICVAPs during removal in children requiring long-term intravenous therapies.

## Methods

### Study population

The institutional review board of Kyungpook National University Hospital approved this study. Between January 2010 and July 2020, a total of 147 cases of TICVAP insertion were performed in 121 patients at Kyungpook National University Hospital. Among these, 98 cases in 72 patients involved TICVAP removal during the same period. Some patients had multiple operations; therefore, one case was counted as a single operation. We retrospectively reviewed the medical records of these 98 cases. The indications were patients requiring chemotherapy, repeated intravenous therapies, or pediatric consultations. After completing the treatment, the TICVAPs were removed by pediatric consultations.

### Technique of TICVAP insertion and removal

All TICVAP insertion and removal procedures were performed by pediatric cardiovascular surgeons in the operating room under general anesthesia. Laryngeal mask airway or mask ventilation was preferred if not contraindicated. Antibiotic prophylaxis was administered upon anesthesia induction for all patients upon insertion and removal. The inserted TICVAPs were the Healthport Venous MiniMAX Polyurethane 5Fr, miniMAX silicone 6.5Fr, or ETI silicone 8Fr (Baxter Healthcare SA, Zurich, Switzerland).

TICVAP insertion was performed through two incisions: one to approach the vein and the other to implant the access port. In the early stage of our experience, vascular access was performed using a traditional visual and palpable anatomic landmarks-guided technique. After blind subclavian vein puncture, the catheter was inserted using the Seldinger technique under the guidance of fluoroscopy. Starting in 2017, we began ultrasound-guided vascular access and insertion of TICVAPs. The patency of the port system was confirmed by backflow of venous blood and injection of a saline solution containing heparin. An anchoring suture from the port to the pectoralis fascia was performed in all implantations. The needle that provides access for infusion of the intravenous drug or fluid was usually inserted in the operating room to keep the site aseptic. The TICVAP was even used on the day of the operation if the child had poor peripheral venous access.

After the completion of treatment, the TICVAP was removed. Undemanding removal was performed through a single incision. Skin incision over the previous access port site, dissection of the access port, and removal of the catheter portion were performed through the same incision site. For complicated removals, if the catheter did not come out easily, a second small skin incision was made at the previous vein approach site. Followed by blunt dissection, the catheter subsequently release or we pull the catheter as much as we can and intentionally cut it; remaining stuck fragment.

### Definitions

Early complications were defined as occurring up to 7 days after TICVAP insertion and late complications thereafter during the indwelling of the TICVAP. Catheter malfunction was defined as no forward flow of saline or backflow of blood upon attempted flushing of the port with heparinized saline despite repeat attempts. Mechanical malfunction included catheter dislodgement, kinking, fracture, embolization, or access port rotation. Thrombotic malfunction included all malfunction, except mechanical malfunction.

### Statistical analysis

Continuous variables are expressed as median and interquartile range for non-normally distributed data. Differences between continuous variables were assessed using the Mann–Whitney U test for non-normally distributed data. Categorical variables are expressed as numbers and percentages. Differences between categorical variables were assessed using Fisher’s exact test. The risk factor analysis of retained fragment of TICVAPs during removal was performed through logistic regression. Receiver operating characteristic (ROC) curves and the area under the curve (AUC) are presented to aid the removal or exchange timing of TICVAPs in order to not result in retained fragment of TICVAPs. Values were considered statistically significant when the p-value was < 0.05. All statistical analyses were performed using R version 4.0 (R Foundation for Statistical Computing, Vienna, Austria, http://www.R-project.org).

## Results

### Characteristics of patients and TICVAPs

A total of 147 cases of TICVAP insertion were performed in 121 patients. Among these patients, 13 were still using their TICVAP at the end of the study period, 15 died with their TICVAP still inserted, 18 were transferred to another hospital, and 3 patients had their TICVAP removed in another hospital. We retrospectively review the medical records of the remaining 72 patients, including 98 cases of insertion and removal at Kyungpook National University Hospital.

Patient demographic characteristics are described in Table 1. The median age and body weight at the time of TICVAP insertion were 77.5 months and 23.1 kg, respectively. The median indwelling duration and body weight change during indwell were 12.5 months and 4.0 kg, respectively. The most common patient diagnoses were solid tumor (35.7%) and acute leukemia (31.6%).

**Table 1:** Characteristics of patient

Characteristics	n = 98
Sex	
Male	63 (64.3%)
Female	35 (35.7%)
Age (months)	
At TICVAP insertion	77.5 (18.0–144.0)
At TICVAP removal	109.5 (48.0–162.0)
Body weight (kg)	
At TICVAP insertion	23.1 (12.5–41.1)
At TICVAP removal	32.0 (17.0–50.0)
Indwell duration of TICVAP	
Cumulative duration (days)	32255
Median duration (months)	12.5 (5.0–37.0)
Body weight change during indwell (kg)	4.0 (1.4–9.7)
Follow-up duration (months)	88.0 (45.0–100.0)
Diagnosis	
Hematologic	
Acute leukemia	31 (31.6%)
Hemophilia	3 (3.1%)
Others^a^	8 (8.2%)
Solid tumor	35 (35.7%)
Non-malignant	11 (11.2%)
Lymphoma	10 (10.2%)

Data are median (IQR) or n (%). IQR, interquartile range; TICVAP, totally implantable central venous access port

^a^Hemophagocytic lymphohistocytosis (3), aplastic anemia (2), factor X deficiency (1), hemolytic anemia (1), myelodysplastic syndrome (1)

The characteristics of TICVAP are described in Table 2. The most common indication for TICVAP insertion was chemotherapy (82.7%), and that for removal was termination of therapies (43.4%). The most common TICVAP insertion site of venous access was the left subclavian vein (LSCV, 71.4%). In the early stage of our experience, the subclavian vein of the non-dominant hand side was the first choice for venous access. Since 2017, we began ultrasound-guided vascular access and insertion of TICVAPs. The venous access was changed to the right internal jugular vein for the initial venous access. Without fluoroscopy, we were unable to check the guidewire and catheter passing through the innominate vein from the LSCV. Therefore, we preferred to use the right internal jugular vein with ultrasound, which is a straight course to reach the right atrium, and there is no need to confirm using fluoroscopy. The most common used catheter size and material were 6.5 Fr (68.4%) and silicon (75.5%), respectively.

**Table 2:** Characteristics of totally implantable central venous access port

Characteristics	n = 98
Insertion indication	
Chemotherapy	81 (82.7%)
Intravenous therapy	13 (13.3%)
Nutritional support	4 (4.1%)
Removal indication	
Termination of therapy	43 (43.4%)
Infection	23 (23.5%)
Bacterial sepsis	10 (10.2%)
Fungal sepsis	6 (6.1%)
Unknown fever focus	6 (6.1%)
Local infection of port site	2 (2%)
Malfunction	6 (6.1%)
Others^a^	4 (40.8%)
Insertion site	
Left subclavian vein	70 (71.4%)
Right subclavian vein	20 (20.4%)
Right internal jugular vein	4 (4.1%)
Left internal jugular vein	3 (3.1%)
Femoral vein	1 (1%)
Catheter size	
5 Fr	24 (24.5%)
6.5 Fr	67 (68.4%)
8 Fr	7 (7.1%)
Catheter material	
Polyurethane	24 (24.5%)
Silicon	74 (75.5%)

Data are n (%). Fr, French

^a^Port site pain or skin color change

TICVAP related complications are described in Table 3. Central line associated blood stream infection (CLABSI, 19.4%) was the most common complication.

**Table 3:** Totally implantable central venous access port related complications

Complications	n = 98
Early complications	
Arterial puncture	2 (2%)
Multiple puncture trial on others site	5 (5%)
Hemopneumothorax	0 (0%)
Thrombotic malfunction	0 (0%)
Mechanical malfunction	0 (0%)
Late complications	
CLABSI	19 (19.4%)
Thrombotic malfunction	5 (5%)
Mechanical malfunction	1 (1%)
Port site problems	3 (3.1%)

Data are n (%). CLABSI: central line associated blood stream infection

### Stuck fragment of TICVAPs during removal

**Fig. 1:**
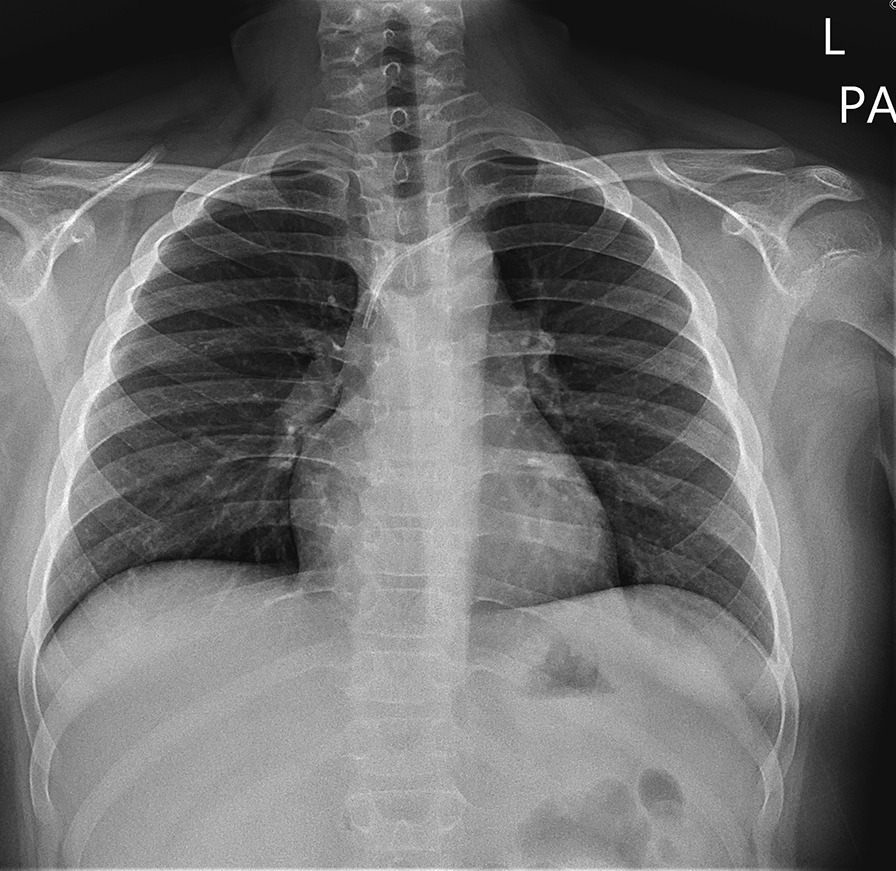
Postoperative chest X-ray showing a patient with a stuck fragment of a totally implantable central venous access port after removal operation

Of the 98 cases, 8 patients had an incomplete removal of their TICVAP, resulting in a stuck fragment of the catheter in the central venous system. Patients were divided into the complete removal (Group N, n = 90) and stuck fragment (Group S, n = 8) groups, and their characteristics were compared in Table 4. In Group S, the catheters were incorporated into the wall of the innominate vein and could not be completely removed (Fig. 1). All children in Group S were male, had acute leukemia, their TICVAPs were used for chemotherapy, and the TICAVPs were removed after chemotherapy. Compared with the complete removal group (Group N), stuck fragments were significantly found in patients diagnosed with acute leukemia compared with other diagnoses in Group S (p < 0.001). Correspondingly, the platelet count at the time of TICVAP insertion was significantly lower in Group S (p < 0.001). Indwelling duration of TICVAP was significantly longer in the S group (p < 0.001). Body weight change during the indwelling of TICVAP was significantly larger in the S group (p < 0.001). Contrary to our expectations, the venous insertion site of TICVAP was insignificant in Group S. The median follow-up duration of Group S after TICVAP removal was 37.0 months. No one in Group S experienced complications related to stuck fragment during the follow-up period.

**Table 4:** Comparison of patient characteristics during totally implantable central venous access ports removal

Characteristics	Complete removal (Group N, n = 90)	Stuck fragment (Group S, n = 8)	p-value
Sex			0.048
Male	55 (61.1%)	8 (100.0%)	
Female	35 (38.9%)	0 (0.0%)	
Age at TICVAP insertion (months)	85.0 [17.0;144.0]	37.5 [31.5;57.0]	0.357
Body weight at TICVAP insertion (kg)	24.6 [11.7;48.0]	17.0 [15.2;19.1]	0.206
Age at TICVAP removal (months)	110.0 [40.0;163.0]	90.0 [84.5;117.5]	0.922
Body weight at TICVAP removal (kg)	32.0 [16.5;50.0]	32.1 [27.1;43.2]	0.704
Indwell duration of TICVAP (months)	10.5 [ 5.0;20.0]	54.5 [52.0;60.0]	< 0.001
Body weight change during indwell (kg)	3.5 [ 1.2; 7.5]	12.9 [12.2;17.6]	< 0.001
Follow-up duration (months)	86.5 [44.0;100.0]	91.0 [80.0;102.5]	0.399
Diagnosis			< 0.001
Acute leukemia	23 (25.6%)	8 (100.0%)	
Remaining diagnoses^a^	67 (74.4%)	0 (0.0%)	
Platelet count at TICVAP insertion (10^3^/ul)	294.5 [182.0;376.0]	71.5 [19.0;102.0]	< 0.001
TICVAP insertion indication			0.713
Chemotherapy	73 (81.1%)	8 (100.0%)	
Intravenous therapies	13 (14.4%)	0 (0.0%)	
Nutritional support	4 (4.4%)	0 (0.0%)	
TICVAP insertion site			0.534
Left subclavian vein	63 (70.0%)	8 (100.0%)	
Right subclavian vein	19 (21.1%)	0 (0.0%)	
Right internal jugular vein	4 (4.4%)	0 (0.0%)	
Left internal jugular vein	3 (3.3%)	0 (0.0%)	
Femoral vein	1 (1.1%)	0 (0.0%)	
TICVAP insertion site			
Left subclavian vein	63 (70.0%)	8 (100.0%)	0.102
Remaining insertion sites^b^	27 (30.0%)	0 (0.0%)	
TICVAP catheter size			0.568
5 Fr	22 (24.4%)	2 (25.0%)	
6.5 Fr	62 (68.9%)	5 (62.5%)	
8 Fr	6 (6.7%)	1 (12.5%)	
TICVAP catheter material			1.000
Polyurethane	22 (24.4%)	2 (25.0%)	
Silicon	68 (75.6%)	6 (75.0%)	

Data are median (IQR) or n (%). IQR, interquartile range; TICVAP, totally implantable central venous access port; Fr, French

^a^Refer to Table 1. ^b^Refer to Table 2 or Table 4

**Fig. 2:**
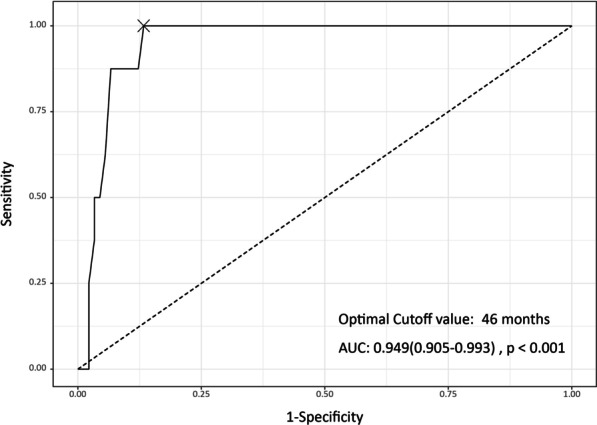
Receiver operating characteristic curve of stuck fragment of totally implantable central venous access ports during removal according to indwell duration of totally implantable central venous access port. The area under the curve was 0.949 (95% Cl, 0.905–0.993), and the optimal cut-off value is 46 months

**Fig. 3:**
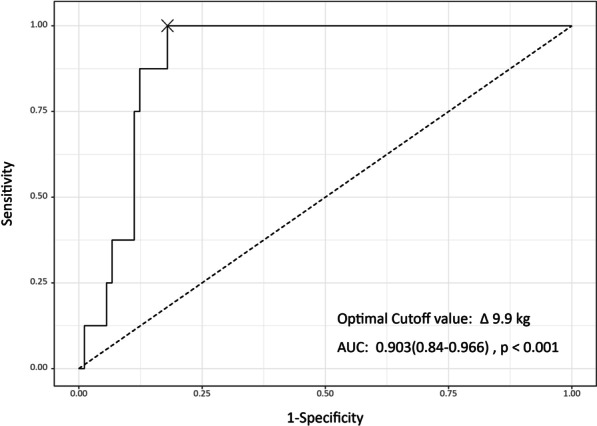
Receiver operating characteristic curve of stuck fragment of totally implantable central venous access ports during removal according to a patient’s body weight change during totally implantable central venous access port indwell. The area under the curve was 0.903 (95% Cl, 0.840–0.966), and the optimal cut-off value was 9.9 kg

Table 5 shows the univariate analysis of the parameters that were associated with stuck fragment during TICVAP removal. Notably, the insertion site of TICVAP was not associated with the presence of a stuck fragment during TICVAP removal. In the multivariate logistic regression, indwell duration of TICVAP (odds ratio [OR], 1.13; 95% confidence interval [Cl] 1.02–1.37, p = 0.10), body weight change during indwell (OR, 1.00; 95% Cl 0.83–1.18, p = 0.97), and platelet count at TICVAP insertion (OR, 0.98; 95% Cl 0.95–0.99; p = 0.48) showed a trend toward an increased risk of stuck fragment of TICVAPs during removal. A patient’s platelet count is related to their diagnosis of acute leukemia and cannot be changed or managed. A patient’s body weight can be measured and the indwell duration of TICVAP can be modulated by early removal and reinsertion. The ROC curve of the stuck fragment of TICVAPs during removal according to the indwell duration of TICVAP is presented in Fig. 2. The AUC was 0.949 (95% Cl, 0.905–0.993, sensitivity 100%, and specificity 86.7%) and the optimal cutoff value was 46 months. The ROC curve of the stuck fragment of TICVAPs during removal according to body weight changes during the TICVAP indwelling is presented in Fig. 3. The AUC was 0.903 (95% Cl 0.84–0.966, sensitivity 100%, and specificity 82%), and the optimal cutoff value was 9.9 kg.

**Table 5:** Risk factors of stuck fragment during totally implantable central venous access ports removal

Parameters	Univariate		Multivariate	
	OR (95% Cl)	p-value	OR (95% Cl)	p-value
Age at TICVAP insertion	0.99 (0.98–1.00)	0.203		
Body weight at TICVAP insertion	0.96 (0.90–1.01)	0.146		
Body weight at TICVAP removal	1.00 (0.96–1.04)	0.841		
Indwell duration of TICVAP	1.12 (1.06–1.25)	0.003	1.13 (1.02–1.37)	0.098
Body weight change during indwell	1.13 (1.05–1.24)	0.003	1.00 (0.83–1.18)	0.969
Platelet count at TICVAP insertion	0.98 (0.96–0.99)	0.005	0.98 (0.95–0.99)	0.048
TICVAP catheter size	1.13 (0.46–2.87)	0.789		
TICVAP catheter material	0.97 (0.21–6.95)	0.972		

CI, confidence interval; TICVAP, totally implantable central venous access port

## Discussion

In our study, the most common complication of TICVAP was CLABSI, which causes morbidity and mortality. It is well-known that biofilm, such as bacterial colonization, is one of the main factors, which is responsible for the onset of CLABSI. Biofilm is formed after 24–48 h following the insertion of TICVAP by the bacteria and plasma, which modifies the metabolism of the bacteria, developing resistance to antibiotics, subsequently interfering with the immune defenses of the human body. The diagnosis of CLABSI is based on clinical and laboratory criteria. Adequate treatment and even more importantly, effective prevention represent to reduce the incidence of CLABSI during the use of TICVAP [6].

Meanwhile, among the many complications of TICVAPs, stuck fragment of the catheter is one of the unsolved complications. Our findings are in line with this notion, as we found that the indwell duration of the catheter was an important risk factor, meaning the longer the catheter remained in the blood vessel, the more likely the catheter was to become adherent. It is important to consider the feasible causes and risk that could result in catheter adherence to the vessel wall. Forauer et al. demonstrated histologic changes in the vein wall adjacent to indwelling central venous catheters. They reported that long-term catheters displayed vein wall thickening and bridges from the vein wall to the catheter [7]. Because all patients in Group S received chemotherapy due to acute leukemia, we presumed that particular chemotherapeutic drugs for acute leukemia may contribute to histologic changes in the vein wall and catheter fixation. Similar results were shown in a study by Wilson’s group [2].

Some authors have emphasized that the material type of the catheter (whether polyurethane or silicone) affects the stuck phenomenon. They suggested that polyurethane catheters are more likely to adhere to the inner wall of the central vein with surrounding calcification than silicone catheters [2, 3, 8, 9]. However, other studies have shown similar results to our study, in that the type of catheter did not seem to affect whether a catheter gets stuck [4, 5, 10]. The influence of catheter material requires further study.

There is also much controversy about the association between the TICVAP venous insertion site and a retained catheter. Numerous studies have reported that LSCV catheterization is associated with more complications, such as catheter fracture, pinch off syndrome, thrombosis, or stuck catheter [11–14]. In our study, the TICVAP venous insertion site of all patients in Group S was LSCV. Before the statistical analysis of this study, we vaguely thought that LSCV access might be a risk factor for stuck fragment of TICVAPs during removal. The location of the stuck fragment during removal in all Group S patients was the entrance site of the catheter introduced through the LSCV between the clavicle and the first rib. Therefore, we thought that when introduced via the subclavian vein, either left or right, a catheter seemed to get stuck when a venous catheter was damaged or disrupted by repeated mechanical compression between the clavicle and the first rib. Hinke et al. also reported a similar mechanism of pinch off syndrome [13]. However, far from our expectations, the TICVAP venous insertion site had no statistical significance with stuck fragment of the catheters.

Additionally, we reviewed the body weight change of patients during TICVAP indwelling. Since the indwell duration of TICVAP was relevant and the TICVAP insertion site was irrelevant, we were of the opinion that a child’s physical growth might affect whether a catheter would get stuck. We found, using multivariant logistic regression, that body weight change during TICVAP indwelling increased the risk of stuck fragment of the TICVAP during removal (Table 5).

There is literature related to the removal of stuck catheters, and various treatment options have been reported. Interventional endovascular retrieval seems to be the most common attempt. There are also reports of the use of various tools, such as a laser beam, introducer sheath, wire, endoluminal balloon dilatation, and Fogarty arterial embolectomy catheter. In groups with aggressive management, they have even attempted open heart surgery, sternotomies, and thoracotomies [2–5, 7, 10, 15–20]. Maizlin et al. reported extensive dissection with resection of the clavicle or extensive venous angioplasty to remove the retained central catheters in four patients [3]. In another group, remnant catheter tips were retained in the vessel and only closely monitored in the outpatient clinic at follow-up [2, 3, 7, 19, 20]. The management of stuck catheters remains controversial, particularly in asymptomatic patients who require more aggressive procedures than TICVAP insertion, which is a simple procedure. Moreover, the latent consequences of a stuck catheter are unclear. Therefore, the decision regarding the treatment direction of an asymptomatic patient with a stuck catheter should be made carefully. The migration or embolization of retained catheter fragments has been described [21–23]. The migration of the catheter fragment suggests that the catheter was not firmly attached to the vessel wall. These retained catheter fragments can be relatively easily removed by appropriate interventional cardiac or radiologic experts. However, if the catheter fragment is firmly fixed to the vessel wall, it is quite possible that a vigorous attempt to remove the adherent catheter could result in a tear in the central vein with consequent severe uncontrolled bleeding.

In our Group S patients, we assumed that the stuck fragment was well integrated with the vessel wall, so we did not consider endovascular retrieval. We also predicted they had little chance of embolism, and because the size of the catheter is small compared to the size of the innominate vein, there was little risk of thrombosis. The stuck fragment of the catheter was in situ, and the intentionally cut off part of the catheter tip might have been in a blood vessel or buried in the muscle layer under the clavicle. We did not use any anticoagulants. No patient experienced a thrombotic or embolic event due to the stuck fragment of the TICVAPs after removal, and none of these patients had catheter-related infections. The follow-up duration after removal operation in Group S was 37.0 months (range, 27.0 to 42.0). We have continued close monitoring of these patients for the occurrence of related complications.

## Conclusion

We suggest a prophylactic catheter exchange before an indwell duration of 46 months and a body weight change up to 9.9 kg to prevent a stuck fragment of the catheter. In particular, this suggestion should be considered in children with rapidly growing acute leukemia. Although there was no statistical significance, it would be clinically useful for the surgeon to keep potential complications in mind when removing catheters that are inserted via the LSCV. The management of stuck fragment remains controversial, specially in asymptomatic patients. In these patients, we suggest careful and close observation rather than aggressive and invasive treatment.

## Data Availability

The datasets used and/or analysed during the current study available from the corresponding author on reasonable request.

## Abbreviations

TICVAP: Totally implantable central venous access port
ROC: Receiver operating characteristic
AUC: Area under the curve
CLABSI: Central line associated blood stream infection
